# New Hepatitis E Virus Genotype in Bactrian Camels, Xinjiang, China, 2013

**DOI:** 10.3201/eid2212.160979

**Published:** 2016-12

**Authors:** Patrick C.Y. Woo, Susanna K.P. Lau, Jade L.L. Teng, Kai-Yuan Cao, Ulrich Wernery, Tony Schountz, Tsz Ho Chiu, Alan K.L. Tsang, Po-Chun Wong, Emily Y.M. Wong, Kwok-Yung Yuen

**Affiliations:** The University of Hong Kong, Hong Kong, China (P.C.Y. Woo, S.K.P. Lau, J.L.L. Teng, T.H. Chiu, A.K.L. Tsang, P.-C. Wong, E.Y.M Wong, K.-Y. Yuen);; Sun Yat-sen University, Guangzhou, China (K.-Y. Cao);; Central Veterinary Research Laboratory, Dubai, United Arab Emirates (U. Wernery);; Colorado State University, Fort Collins, Colorado, USA (T. Schountz)

**Keywords:** hepatitis E virus, Bactrian camel, Camelus bactrianus, new genotype, China, viruses, genotype, epidemiology, Old World camelid species, genomic analysis, zoonoses, transmission, humans, camel food products, genus Orthohepevirus, family Hepeviridae

**To the Editor:** Hepatitis E virus (HEV) is a member of the family *Hepeviridae*, genus *Orthohepevirus*, which comprises 4 species, *Orthohepevirus A–D*. *Orthohepevirus A* contains 7 genotypes (HEV1–7) (*1**,**2*). HEV1 and HEV2 infect humans only; HEV3, HEV4, and HEV7 can infect humans and other mammals; and HEV5 and HEV6 have been detected in animals only. 

Worldwide, HEV is the most common cause of acute viral hepatitis in humans. The disease is generally self-limiting, but high death rates have been observed among HEV-infected pregnant women. Chronic HEV infection is a problem in immunocompromised patients, such as solid organ transplant recipients (*3*). Human HEV3 and HEV4 infections have been associated with consumption of undercooked pork or game meat *(**4**)*.

In 2014, we described the discovery of a novel genotype of HEV in dromedaries (*Camelus dromedarius* or 1-humped camels), suggesting another possible source of human HEV infection (*5*). This dromedary HEV was subsequently classified as a novel *Orthohepevirus A* genotype, HEV7 (*1**,**2*). Recently, this HEV7 genotype was also isolated from a liver transplant recipient from the Middle East with chronic HEV infection (*6*). The patient regularly consumed dromedary camel meat and milk, implying camel-to-human transmission of the virus (*6*). 

Like the dromedary, the Bactrian camel (*Camelus bactrianus* or 2-humped camels) is an Old World camelid species. Thus, we hypothesize that Bactrian camels may also be reservoirs of HEV. To test this hypothesis and increase our understanding of the epidemiology of HEV in camels, we performed a molecular epidemiology study using feces samples from camels in China.

During November 2012–May 2013, we collected and tested 1 feces sample each from 205 Bactrian camels on a farm in Xinjiang, China. We performed RNA extraction and reverse transcription PCR (RT-PCR) as previously described (*7*). We screened for HEV by PCR amplification of a 251-bp fragment of open-reading frame (ORF) 2, using primers 5′-GTTGTCTCAGCCAATGGCGA-3′ and 5′-GTAGTTTGGTCATACTCAGCAGC-3′. PCR was performed, using previously described conditions (*7*), with the annealing temperature set at 50°C. DNA sequencing and quantitative real-time RT-PCR were performed as previously described (*7*). Three samples were positive for HEV; we performed complete genome sequencing of these samples as described (Technical Appendix) (*5**,**7*). We also performed comparative genomic analysis as previously described (*1**,**2**,**8*). We constructed a phylogenetic tree using the maximum-likelihood method and MEGA7 (*9*); bootstrap values were calculated from 1,000 trees. The optimal substitution model for each ORF was selected by MEGA7 (Figure).

**Figure:**
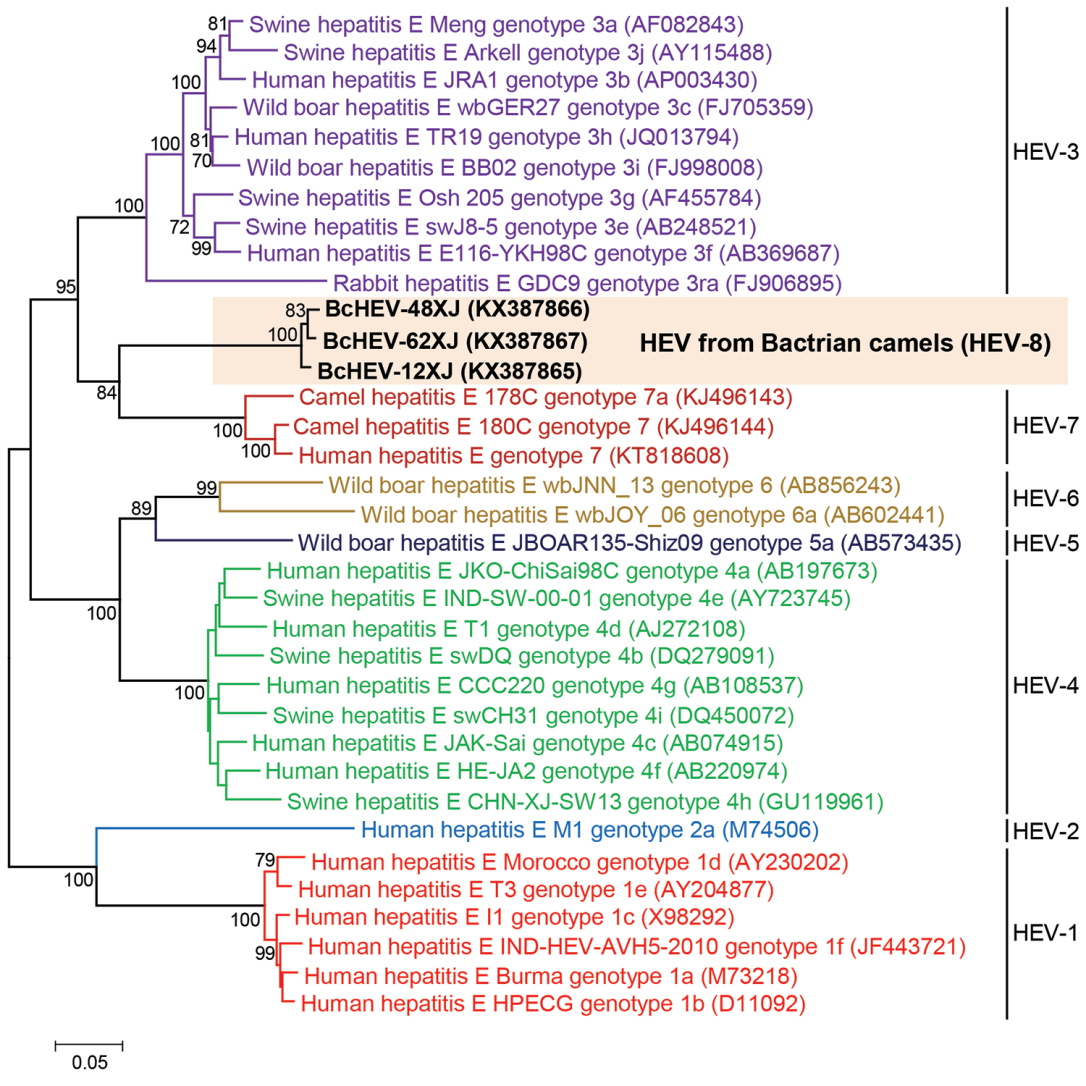
Phylogenetic analyses of the proteins of concatenated ORF1/ORF2, excluding the hypervariable region, of Bactrian camel hepatitis E virus (HEV) and other HEV genotypes (HEV1–HEV7) within the species *Orthohepevirus A* (family *Hepeviridae*). The tree was constructed using the maximum-likelihood method using the Jones–Taylor–Thornton substitution model with invariant sites and gamma distributed rate variation. The analysis included 2,282 amino acid positions (aa residues 1–706 and 789–2409, numbered with reference to GenBank sequence M73218). Bold indicates the 3 strains of BcHEV with complete genomes sequenced in this study. GenBank accession numbers are shown in parentheses. Scale bar indicates the estimated number of substitutions per 20 aa. ORF, open-reading frame.

RT-PCR for a 251-bp fragment in ORF2 of HEV was positive for 3 (1.5%) of the 205 fecal samples; virus loads were 1.6 × 10^3^, 2.1 × 10^3^, and 1.8 × 10^4^ copies/mg, respectively. Whole-genome sequencing of the 3 Bactrian camel HEV (BcHEV) strains (GenBank accession nos. KX387865–7) showed genome sizes of 7,212–7,223 bp and a G + C content of 52.7%–53.1%. Overall, nucleotides in the BcHEV genome differed by >20% compared with those in all other HEVs. Genomes of the 3 BcHEV isolates contained 3 major ORFs; genome organization was typical of and characteristics were similar to those of HEVs from other *Orthohepevirus A* species. Phylogenetic trees constructed using ORF1, ORF2, ORF3, and concatenated ORF1/ORF2, excluding the hypervariable region, showed that these 3 BcHEV isolates clustered with the 2 dromedary camel HEV7 strains and the HEV7 strain from the liver-transplant recipient with chronic hepatitis (Figure; Technical Appendix Figure 1) (*5**,**6*). However, amino acid distances based on the concatenated ORF1/ORF2, excluding the hypervariable region of the 3 BcHEV isolates and the existing genotypes, ranged from 0.095 to 0.148, which was greater than the threshold (p-distance = 0.088) to demarcate intergenotype distance (*1**,**2*). Using this criterion, we propose that the 3 BcHEV isolates should constitute a new HEV genotype, HEV8.

A recent study in Dubai, United Arab Emirates, showed that HEV accounted for 40% of acute hepatitis cases (*10*). Even though HEV is an emerging pathogen in the Middle East, limited sequence data exist regarding the virus on the Arabian Peninsula. Recently, we discovered the HEV7 genotype in 1.5% of 203 feces samples from dromedaries in Dubai (*5*). In the current study, we detected a new HEV genotype in 1.5% of 205 Bactrian camels on a farm in Xinjiang. Comparative genomic and phylogenetic analyses showed that BcHEV represents a previously unrecognized HEV genotype. It has been shown that HEV7 from dromedaries can be transmitted to humans; thus, meat and milk from Bactrian camels might pose a similar risk to humans. The increasing discoveries of camel viruses and of their transmission to humans highlight the need for caution when handling these mammals and processing food products derived from them.

Technical Appendix Complete genome sequencing of the 3 Bactrian camel hepatitis E virus (HEV) isolates from a study of HEV genotypes in Bactrian camels, Xinjiang, China, 2013.
