# Supplementary material for: New Hepatitis E Virus Genotype in Bactrian Camels, Xinjiang, China, 2013
Source: Emerg Infect Dis. 2016 Dec;22(12):2219–21. doi: 10.3201/eid2212.160979 (PMC5189163; doi:10.3201/eid2212.160979)
Supplement: Technical Appendix — Complete genome sequencing of the 3 Bactrian camel hepatitis E virus (HEV) isolates from a study of HEV genotypes in Bactrian camels, Xinjiang, China, 2013. [file 16-0979-Techapp-s1.pdf]

Article DOI: http://dx.doi.org/10.3201/eid2212.160979

New Hepatitis E Virus Genotype in Bactrian Camels, Xinjiang, China, 2013

Technical Appendix

Complete Genome Sequencing

Three complete genomes of Bactrian camel HEV (BcHEV) strains, including BcHEV-12XJ, BcHEV-48XJ and BcHEV-62XJ, were amplified and sequenced using the RNA extracted from the original specimens as templates. The RNA was converted to cDNA by a combined random-priming and oligo(dT) priming strategy. The cDNA was amplified by primers designed by multiple alignments of the genomes of other HEVs with complete genomes available. Additional primers were designed from the results of the first and subsequent rounds of sequencing (Table). The 5′ ends of the viral genomes were confirmed by rapid amplification of cDNA ends using the 5′/3′ RACE kit (Roche, Germany). Sequences were assembled and manually edited to produce final sequences of the viral genomes.

Technical Appendix Table. Primers for amplification of the three BcHEV genomes

Primers	5′ to 3′
Forward	
LPW28520	GTTGTCTCAGCCAATGGCGA
LPW28892	CGAAGGCTTACGAATGTTGC
LPW29061	ATCCGTTGGTCATTGAGA
LPW29066	ACTGTTGAGCTTACAGTTG
LPW29070	TGCATGGTGTTTGAGAATGA
LPW31223	CCGGCCCCTACAGTCTTTCATAT
LPW31225	CCGCTAATCCTGGTGCTATTA
LPW29072	TGCTGGACTTGACTAACTCA
LPW31228	GTATTGCCTCTGAACTTGT
LPW31226	GTACGAAGCTGTATGAAGCTGCTCA
LPW31432	GAAGGGTCTGAGGTCGATT
LPW31299	GGCTGTACTGTTGCTGTTCCT
LPW32175	CCATGTGTGGGAGTCCAA
Reverse	
LPW28521	GTAGTTTGGTCATACTCAGCAGC
LPW28893	CTGAGAATCAACCCGGTCA
LPW29062	CAACTGTAAGCTCAACAGT
LPW29067	CGAGTGAGTGCAACAATAGCA
LPW29071	GCTGAGAATCAACCCGGTCA
LPW31231	CTTACCAGAACCAGGGACA
LPW31233	CAGAACCCTTTCAGAGACTCCTT
LPW28521	GTAGTTTGGTCATACTCAGCAGC
LPW29077	GCCCTGAGTGTAATTCTCTT
LPW31233	CAGAACCCTTTCAGAGACTCCTT

LPW31433	GCATGTGCACGAGAAGATT
LPW32181	GCATAATTGGACGCCTCAG
LPW31433	GCATGTGCACGAGAAGATT

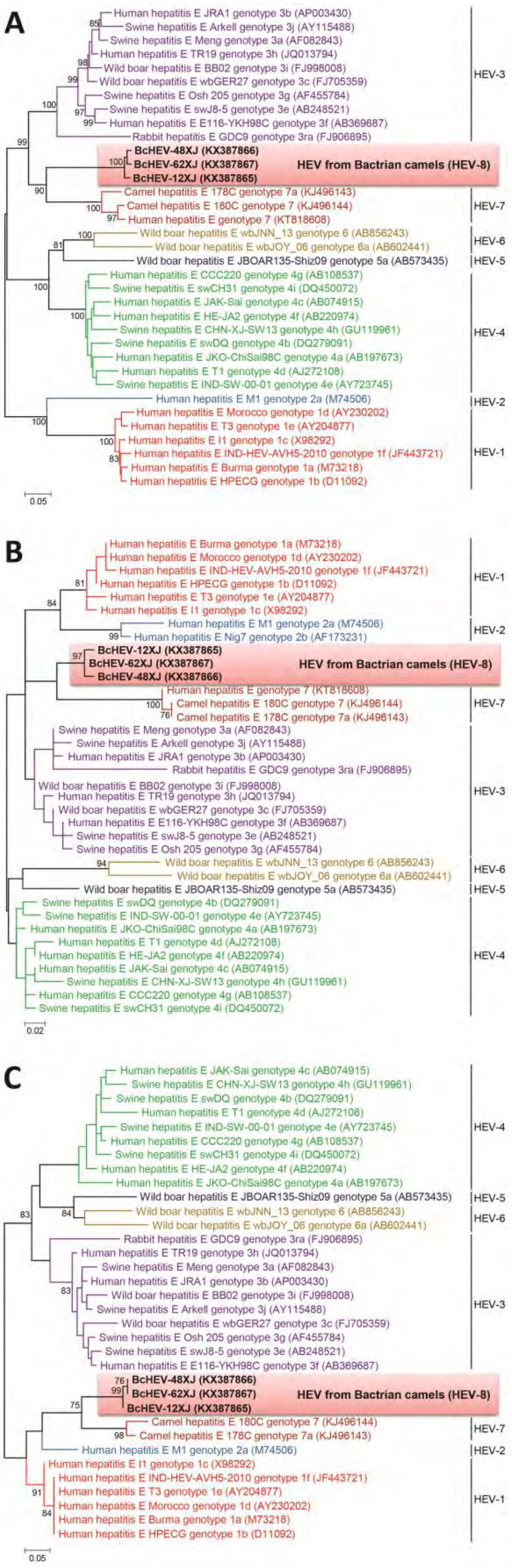
A
Human hepatitis E JRA1 genotype 3b (AP003430)
Swine hepatitis E Arkell genotype 3j (AY115488)
Swine hepatitis E Meng genotype 3a (AF082843)
Human hepatitis E TR19 genotype 3h (JQ013794)
Wild boar hepatitis E BB02 genotype 3i (FJ998008)
Wild boar hepatitis E wbGER27 genotype 3c (FJ705359)
Swine hepatitis E Osh 205 genotype 3g (AF455784)
Swine hepatitis E swJ8-5 genotype 3e (AB248521)
Human hepatitis E E116-YKH98C genotype 3f (AB369687)
Rabbit hepatitis E GDC9 genotype 3ra (FJ906895)
HEV-3
BcHEV-48XJ (KX387866)
BcHEV-62XJ (KX387867)
BcHEV-12XJ (KX387865)
HEV from Bactrian camels (HEV-8)
Camel hepatitis E 178C genotype 7a (KJ496143)
Camel hepatitis E 180C genotype 7 (KJ496144)
Human hepatitis E genotype 7 (KT818608)
HEV-7
Wild boar hepatitis E wbJNN_13 genotype 6 (AB856243)
Wild boar hepatitis E wbJOY_06 genotype 6a (AB602441)
HEV-6
Wild boar hepatitis E JBOAR135-Shiz09 genotype 5a (AB573435)
HEV-5
Human hepatitis E CCC220 genotype 4g (AB108537)
Swine hepatitis E swCH31 genotype 4i (DQ450072)
Human hepatitis E JAK-Sai genotype 4c (AB074915)
Human hepatitis E HE-JA2 genotype 4f (AB220974)
Swine hepatitis E CHN-XJ-SW13 genotype 4h (GU119961)
Swine hepatitis E swDQ genotype 4b (DQ279091)
Human hepatitis E JKO-ChiSai98C genotype 4a (AB197673)
Human hepatitis E T1 genotype 4d (AJ272108)
Swine hepatitis E IND-SW-00-01 genotype 4e (AY723745)
HEV-4
Human hepatitis E M1 genotype 2a (M74506)
HEV-2
Human hepatitis E Morocco genotype 1d (AY230202)
Human hepatitis E T3 genotype 1e (AY204877)
Human hepatitis E I1 genotype 1c (X98292)
Human hepatitis E IND-HEV-AVH5-2010 genotype 1f (JF443721)
Human hepatitis E Burma genotype 1a (M73218)
Human hepatitis E HPECG genotype 1b (D11092)
HEV-1
0.05
B
Human hepatitis E Burma genotype 1a (M73218)
Human hepatitis E Morocco genotype 1d (AY230202)
Human hepatitis E IND-HEV-AVH5-2010 genotype 1f (JF443721)
Human hepatitis E HPECG genotype 1b (D11092)
Human hepatitis E T3 genotype 1e (AY204877)
Human hepatitis E I1 genotype 1c (X98292)
HEV-1
Human hepatitis E M1 genotype 2a (M74506)
Human hepatitis E Nig7 genotype 2b (AF173231)
HEV-2
BcHEV-12XJ (KX387865)
BcHEV-62XJ (KX387867)
BcHEV-48XJ (KX387866)
HEV from Bactrian camels (HEV-8)
Human hepatitis E genotype 7 (KT818608)
Camel hepatitis E 180C genotype 7 (KJ496144)
Camel hepatitis E 178C genotype 7a (KJ496143)
HEV-7
Swine hepatitis E Meng genotype 3a (AF082843)
Swine hepatitis E Arkell genotype 3j (AY115488)
Human hepatitis E JRA1 genotype 3b (AP003430)
Rabbit hepatitis E GDC9 genotype 3ra (FJ906895)
Wild boar hepatitis E BB02 genotype 3i (FJ998008)
Human hepatitis E TR19 genotype 3h (JQ013794)
Wild boar hepatitis E wbGER27 genotype 3c (FJ705359)
Human hepatitis E E116-YKH98C genotype 3f (AB369687)
Swine hepatitis E swJ8-5 genotype 3e (AB248521)
Swine hepatitis E Osh 205 genotype 3g (AF455784)
HEV-3
Wild boar hepatitis E wbJNN_13 genotype 6 (AB856243)
Wild boar hepatitis E wbJOY_06 genotype 6a (AB602441)
HEV-6
Wild boar hepatitis E JBOAR135-Shiz09 genotype 5a (AB573435)
HEV-5
Swine hepatitis E swDQ genotype 4b (DQ279091)
Swine hepatitis E IND-SW-00-01 genotype 4e (AY723745)
Human hepatitis E JKO-ChiSai98C genotype 4a (AB197673)
Human hepatitis E T1 genotype 4d (AJ272108)
Human hepatitis E HE-JA2 genotype 4f (AB220974)
Human hepatitis E JAK-Sai genotype 4c (AB074915)
Swine hepatitis E CHN-XJ-SW13 genotype 4h (GU119961)
Human hepatitis E CCC220 genotype 4g (AB108537)
Swine hepatitis E swCH31 genotype 4i (DQ450072)
HEV-4
0.02
C
Human hepatitis E JAK-Sai genotype 4c (AB074915)
Swine hepatitis E CHN-XJ-SW13 genotype 4h (GU119961)
Swine hepatitis E swDQ genotype 4b (DQ279091)
Human hepatitis E T1 genotype 4d (AJ272108)
Swine hepatitis E IND-SW-00-01 genotype 4e (AY723745)
Human hepatitis E CCC220 genotype 4g (AB108537)
Swine hepatitis E swCH31 genotype 4i (DQ450072)
Human hepatitis E HE-JA2 genotype 4f (AB220974)
Human hepatitis E JKO-ChiSai98C genotype 4a (AB197673)
HEV-4
Wild boar hepatitis E JBOAR135-Shiz09 genotype 5a (AB573435)
HEV-5
Wild boar hepatitis E wbJNN_13 genotype 6 (AB856243)
Wild boar hepatitis E wbJOY_06 genotype 6a (AB602441)
HEV-6
Rabbit hepatitis E GDC9 genotype 3ra (FJ906895)
Human hepatitis E TR19 genotype 3h (JQ013794)
Swine hepatitis E Meng genotype 3a (AF082843)
Human hepatitis E JRA1 genotype 3b (AP003430)
Wild boar hepatitis E BB02 genotype 3i (FJ998008)
Swine hepatitis E Arkell genotype 3j (AY115488)
Wild boar hepatitis E wbGER27 genotype 3c (FJ705359)
Swine hepatitis E Osh 205 genotype 3g (AF455784)
Swine hepatitis E swJ8-5 genotype 3e (AB248521)
Human hepatitis E E116-YKH98C genotype 3f (AB369687)
HEV-3
BcHEV-48XJ (KX387866)
BcHEV-62XJ (KX387867)
BcHEV-12XJ (KX387865)
HEV from Bactrian camels (HEV-8)
Camel hepatitis E 180C genotype 7 (KJ496144)
Camel hepatitis E 178C genotype 7a (KJ496143)
HEV-7
Human hepatitis E M1 genotype 2a (M74506)
HEV-2
Human hepatitis E I1 genotype 1c (X98292)
Human hepatitis E IND-HEV-AVH5-2010 genotype 1f (JF443721)
Human hepatitis E T3 genotype 1e (AY204877)
Human hepatitis E Morocco genotype 1d (AY230202)
Human hepatitis E Burma genotype 1a (M73218)
Human hepatitis E HPECG genotype 1b (D11092)
HEV-1
0.05

Technical Appendix Figure. Phylogenetic analyses of A) ORF1, B) ORF2, and C) ORF3 and other genotypes of HEVs (HEV-1 to HEV-7) within the species *Orthohepevirus A*. The trees were constructed using maximum likelihood method and the optimal substitution models of JTT+G+I+F, JTT+G+I and JTT+G were used for ORF1, ORF2 and ORF3, respectively. Amino acid residues 1–1743, 1–660 and 10–123 in ORF1, ORF2 and ORF3, numbered with reference to GenBank sequence M73218, were included in the analyses. For ORF1 and ORF3, the scale bars indicate the estimated number of substitutions per 20 aa. For ORF2, the scale bar indicates the estimated number of substitutions per 50 aa. The three strains of DcHEV with complete genomes sequenced in this study are in bold.
